# Amlexanox Blocks the Interaction between S100A4 and Epidermal Growth Factor and Inhibits Cell Proliferation

**DOI:** 10.1371/journal.pone.0161663

**Published:** 2016-08-25

**Authors:** Ching Chang Cho, Ruey-Hwang Chou, Chin Yu

**Affiliations:** 1 Department of Chemistry, National Tsing Hua University, Hsinchu, Taiwan; 2 Graduate Institute of Cancer Biology and Center for Molecular Medicine, China Medical University, Taichung, Taiwan; 3 Department of Biotechnology, Asia University, Taichung, Taiwan; Russian Academy of Medical Sciences, RUSSIAN FEDERATION

## Abstract

The human S100A4 protein binds calcium, resulting in a change in its conformation to promote the interaction with its target protein. Human epidermal growth factor (EGF) is the target protein of S100A4 and a critical ligand of the receptor EGFR. The EGF/EGFR system promotes cell survival, differentiation, and growth by activating several signaling pathways. Amlexanox is an anti-inflammatory and anti-allergic drug that is used to treat recurrent aphthous ulcers. In the present study, we determined that amlexanox interacts with S100A4 using heteronuclear single quantum correlation titration. We elucidated the interactions of S100A4 with EGF and amlexanox using fluorescence and nuclear magnetic resonance spectroscopy. We generated two binary models (for the S100A4-EGF and S100A4-amlexanox complexes) and observed that amlexanox and EGF share a similar binding region in mS100A4. We also used a WST-1 assay to investigate the bioactivity of S100A4, EGF, and amlexanox, and found that amlexanox blocks the binding between S100A4 and EGF, and is therefore useful for the development of new anti-proliferation drugs.

## Introduction

The human S100A4 protein carries two calcium ion (Ca^2+^)-binding EF-hand motifs. S100A4 has intra- and extracellular biological functions that are related to Ca-induced conformational changes and cell bioactivity[1, 2]. S100A4 proteins are expressed in various diseases, including cancer, vascular disorders, rheumatoid arthritis, and pulmonary disorders [3, 4]; and S100A4 was found to be up-regulated after cardiac infarction and neural injury [5, 6]. Furthermore, S100A4 participates in cell metastasis and tumor cell proliferation. Increased S100A4 expression has been identified in different human cancers [7, 8], and previous reports indicated a relationship between the expression of S100A4 and poor prognosis [9, 10]. In previous research, the S100A4 protein interacted with various ligands, including non-muscle myosin IIA [11], tropomyosin [12], liprin b1 [13], and the tumor suppressor protein p53 [14]. Previous studies indicated that extracellular S100A4 stimulates cell proliferation, invasion, motility, survival, differentiation, and angiogenesis [15, 16]. Epidermal growth factor (EGF) is a hormone that was originally discovered in many human tissues, including the parotid and submandibular glands [17]. EGF promotes cellular survival, differentiation, and proliferation. The biological functions of EGF include the stimulation of DNA synthesis and participating in the curing of gastroesophageal and oral ulcers [18, 19]. The activity of EGF family members is mediated by the EGFR/ErbB receptor tyrosine kinases. A previous report indicated that S100A4 is associated with several ligands of EGFR, such as EGF, heparin-binding epidermal growth factor, betacellulin, and amphiregulin [20]. The EGF/EGFR system promotes cell survival, differentiation, and growth via the activation of several integrated signaling pathways [21].

Amlexanox is an anti-allergic immunomodulator used to heal aphthous ulcers. Amlexanox can bind S100A13 and inhibit the heat shock-induced release of fibroblast growth factor-1 [22, 23]. Amlexanox induces tyrosine phosphorylation of cortactin, which may reversibly inhibit cell migration, induce Src-dependent cell proliferation, and depolymerize actin filaments [24]. HSQC titration is a suitable method for identifying interfaces of protein-ligand or protein-protein interactions [25]. This approach provides useful information for characterizing the exchange regime of binding [26, 27]. In the current report, we used heteronuclear single quantum correlation (HSQC) titration to investigate the interactions between a mutant S100A4 and amlexanox, and between a mutant S100A4 and EGF, and constructed the two High Ambiguity-Driven Docking (HADDOCK) models. The binding affinity between the mutant S100A4 and EGF was determined via fluorescence spectroscopy. A WST-1 cell proliferation assay was used to study the biological activity of S100A4, EGF, and amlexanox. We investigated two complex structure models using various biophysical techniques to characterize the interactions of S100A4 with EGF and amlexanox at the molecular level. The binding interface of amlexanox is similar to the EGF-binding site of S100A4, indicating that this molecule blocks EGF binding to S100A4 and inhibits the S100A4-EGF-mediated induction of EGFR signaling. This study may be useful for drug development, which can inhibit the diseases related to S100A4 and EGF.

## Materials and Methods

### Reagents and chemicals

D_2_O and ^15^NH_4_Cl were used for NMR HSQC spectroscopy and were obtained from Cambridge Isotope Laboratories. Amlexanox was purchased from Sigma-Aldrich. All other chemicals used in this study were of analytical grade.

### Expression and purification of mutant S100A4 (mS100A4) and EGF

S100A4 contains four free cysteine residues. We typically add 5 mM dithiothreitol (DTT) to the buffer conditions for NMR experiments as a reducing agent. However, when complex formation between S100A4 and EGF is studied using NMR, DTT should be avoided because EGF contains three disulfide bonds. We overcame this problem by creating a mutant of S100A4. All four cysteines were mutated to serines (C3S, C76S, C81S, and C86S). In this case, NMR could be carried out without the use of DTT as a reducing agent. The 3D structure of this mutant (mS100A4) is similar to that of the wild-type protein [28]. The cDNA of the quadruple mutant S100A4 (Cys3, Cys76, Cys81, and Cys86) was purchased from Mission Biotech Company. Using pET21b as a vector, this gene was transformed and over-expressed in *Escherichia coli* BL21 (DE3) (Novagen). ^15^N-labeled samples were prepared using ^15^NH_4_Cl as the nitrogen source, and were grown in M9 medium. The cells were cultured at 310 K until the optical density at 600 nm (OD_600_) reached 0.7. The culture was then induced using 1 mM isopropyl β-d-thiogalactopyranoside at 298 K. After 16 h of overnight induction, the cells were centrifuged for 30 min at 4,307 *g*, followed by resuspension in a buffer containing 20 mM Tris-HCl, pH 7.5. The cells were then lysed using a sonicator. A salting-out buffer (50 mM Tris pH 7.5, 300 mM KCl, 1 mM DTT, 1 mM ethylenediaminetetraacetic acid, 10% glycerol, and 4.32 M (NH_4_)_2_SO_4_) was added until the buffer contained 2.16 M (NH_4_)_2_SO_4_, and the samples were maintained at 4°C for 12 h. The cell lysate was removed by centrifugation at 30,624 *g* and 277 K for 45 min. The supernatant was then purified by passage through a Hi-Prep Q XL 16/10 column and washed with an equilibrating buffer (20 mM Tris-HCl, pH 7.5). The protein was then eluted using elution buffer (20 mM Tris-HCl, 1.6 M NaCl, pH 7.5). The mS100A4-containing fraction was further purified using Hi-Prep Phenyl FF 16/10, by first exchanging the buffer for the loading buffer (20 mM Tris-HCl, 2 mM CaCl_2_, pH 7.5), which allows mS100A4 to bind to the column while washing out unwanted impurities. Elution buffer (20 mM Tris-HCl and 20 mM ethylenediaminetetraacetic acid, pH 7.5) was used to detach the target protein. Finally, we used a Hi-load 16/60 Superdex 75 column as a finishing column for final purification and buffer exchange.

The recombinant EGF was fused to the pET-32a (+) vector constructed into BL21 (DE3) Origami B competent cells (Novagen). We cultured the cells and purified the protein as reported previously [29]. Both proteins were estimated to have over 95% purity using sodium dodecyl sulfate-polyacrylamide gel electrophoresis analysis, and the molecular weights were established via mass spectrometry.

### NMR chemical shift assignments

NMR experiments were performed on a Varian 700 MHz NMR spectrometer operating at 298 K with a cryogenic probe. The NMR shift assignments for EGF were reported by Huang et al [29]. All of the chemical shifts of the mutant mS100A4 were assigned previously and deposited in Biological Magnetic Resonance Bank (BMRB). The assignments of mS100A4 were examined in 25 mM Tris-HCl, 100 mM NaCl, and 5 mM CaCl_2_, and ^1^H-^15^N HSQC, HNCOCA, HNCA, CBCACONH HNCACB, and HCCCONH, CCCONH, HBHACONH, HCCH-TOCSY and ^15^N-edited TOCSY-HSQC were performed. We processed the NMR data using VnmrJ 2.3 and analyzed the data using Sparky 3.1 [30].

### Chemical shift perturbation experiments

NMR HSQC experiments were performed on a Varian 700 NMR spectrometer at 298 K with a cryogenic cooled probe. All titrations were performed under the same buffer conditions (25 mM Tris-HCl, 100 mM NaCl, 5 mM CaCl_2_, pH 7.5, and 10% D_2_O). Unlabeled Ca-bound mS100A4 was uniformly added to ^15^N-labeled EGF at molar ratios of 1:0, 1:0.33, 1:0.66, and 1:1. A reverse titration experiment for ^15^N-labeled mS100A4 was performed at molar ratios of 1:0, 1:0.33, 1:0.66, and 1:1. Finally, the titration of mS100A4 and amlexanox was performed at molar ratios of 1:0, 1:0.5, 1:1, 1:2, and 1:3. By overlapping the HSQC titration spectrum, we could calculate the level of intensity reduction or chemical shift perturbation of each peak.

### Fluorescence experiments

We collected florescence experiments using Hitachi F-2500 fluorescence spectroscopy. The tryptophan of EGF showed absorption at 295 nm and maximal emission in the range of 310 to 415 nm. Increasing concentrations of mS100A4 (0–22 μM) were added to EGF (8 μM). The changes in the emission spectrum were plotted as *1/[mS100A4]* versus *1/ (I*_*0*_*–I)* and represented in the following equation [31]:

1/(I0–I)=1/(I0–I)+K/1/(I0–I)·1/[mS100A4]

*I*_*0*,_ *I*, and *I*_*1*_ represent the intensities of emission without mS100A4, at the middle concentration of mS100A4 and at the maximal concentration of mS100A4, and K indicates the dissociation constant.

### WST-1 cell proliferation assay

WST-1 cell proliferation assay was performed according to the manual (Roche). Cells were cultured to the logarithmic growth phase, and then trypsinized and seeded at a density of 1x104 cells/well in a 96-well plate in the day before experiments. Subsequently, cells were incubated in the serum-free medium containing 0.1% BSA for 24h. The serum-starved cells were treated with or without indicated concentrations of recombinant proteins or drugs for another 48h. Before harvest, 1/10 volume of WST-1 (4-[3-(4-iodophenyl)-2-(4-nitrophenyl)-2H-5-tetrazolio]-1,3-benzene disulfonate) cell proliferation reagent (Roche) was added into each well and cells were incubated at 37 oC for another 4h. The medium in the cell culture plate was mixed by gentle agitation on a shaker for 10 min. The absorbance was measured at 450 nm with the Synergy 2 microplate reader (BioTek Instruments, Inc.). The relative cell numbers were determined by the relative absorbance comparing to that from control treatment [32, 33].

### Molecular docking

We docked mS100A4 and EGF (or amlexanox) to obtain an mS100A4-EGF (or mS100A4-amlexanox) tetrameric model via HADDOCK [34]. The NMR coordinates of mS100A4 were obtained from the Protein Data Bank (ID: 2MRD). The NMR coordinates of EGF were also obtained from the Protein Data Bank (ID:2KV4), and the input data for amlexanox were obtained from DrugBank (ID: DB01025). The cross peaks with significant chemical shift perturbations and decreasing intensity were defined as ambiguous interaction constraints of the binding interface of the mS100A4-EGF complex. We defined the active or passive residues via NACCESS [35]. We selected the residues with relative available solvent surface accessible areas of more than 30% for the backbone and side chain as active sites and the residues with values of less than 30% as passive sites. After calculation, the 2,000 rigid body docking models were executed by utilizing HADDOCK and induced optimized potentials for liquid simulation (OPLSX) parameters [36]. Two hundred lower energy structures were optimized by water refinement and used for analysis. The structural representations were generated using PyMOL [37].

## Results

### Fluorescence measurements of proteins containing intrinsic tryptophan

**Fig 1:**
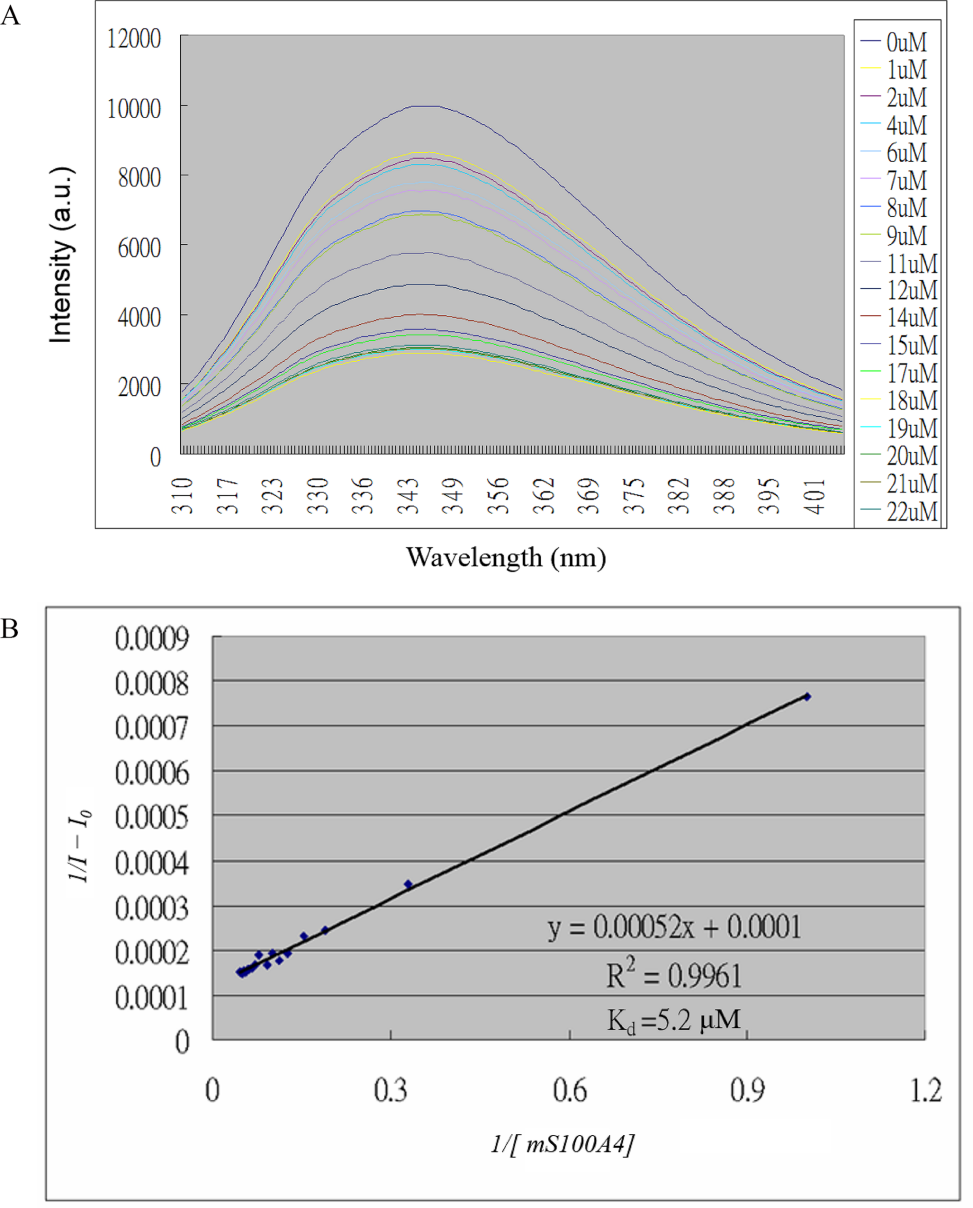
Interactions of EGF with mS100A4 monitored using fluorescence spectroscopy. (A) Fluorescence emission spectra of EGF illustrating the changes in the intrinsic tryptophan fluorescence that occur with increasing concentrations of mS100A4 in the micromolar range. (B) Changes in the fluorescence intensities measured at 345 nm as a function of the mS100A4 concentration were calculated according to Eq 1. The binding was fitted (solid red line) to a one-site binding model.

The emission of protein tryptophan fluorescence was sensitive to conformational changes and the polar environment of the protein when the substrate was associated with the protein. When tryptophan was excited at 295 nm, the proteins displayed maximal emission in the range of 345 to 360 nm in high-polarity environments. Tryptophan residues exhibit maximal emission ranging from 330 to 345 nm in hydrophobic environments (28). EGF contains two tryptophan residues at positions 49 and 50, and no tryptophan residues are present in the mutant S100A4. The HSQC titrations of EGF with mS100A4 indicated that these two tryptophan residues (W49 and W50) interact with mS100A4. Furthermore, EGF displays maximal emission at 345 nm and excitation at 295 nm. The intensity change of the EGF emission at 345 nm was excited at 295 nm. The titration of EGF with mS100A4 caused a decrease in the fluorescence emission of EGF (Fig 1A). We plotted the intensity change of emission versus the concentration of mS100A4, as shown in Fig 1B. The dissociation constant (Kd) is 5.2 μM, which shows the binding affinity of mS100A4 for EGF. The value was in the micromolar range, which also indicated the stability and formation of the complex.

### Mapping the interface between mS100A4 and EGF

**Fig 2:**
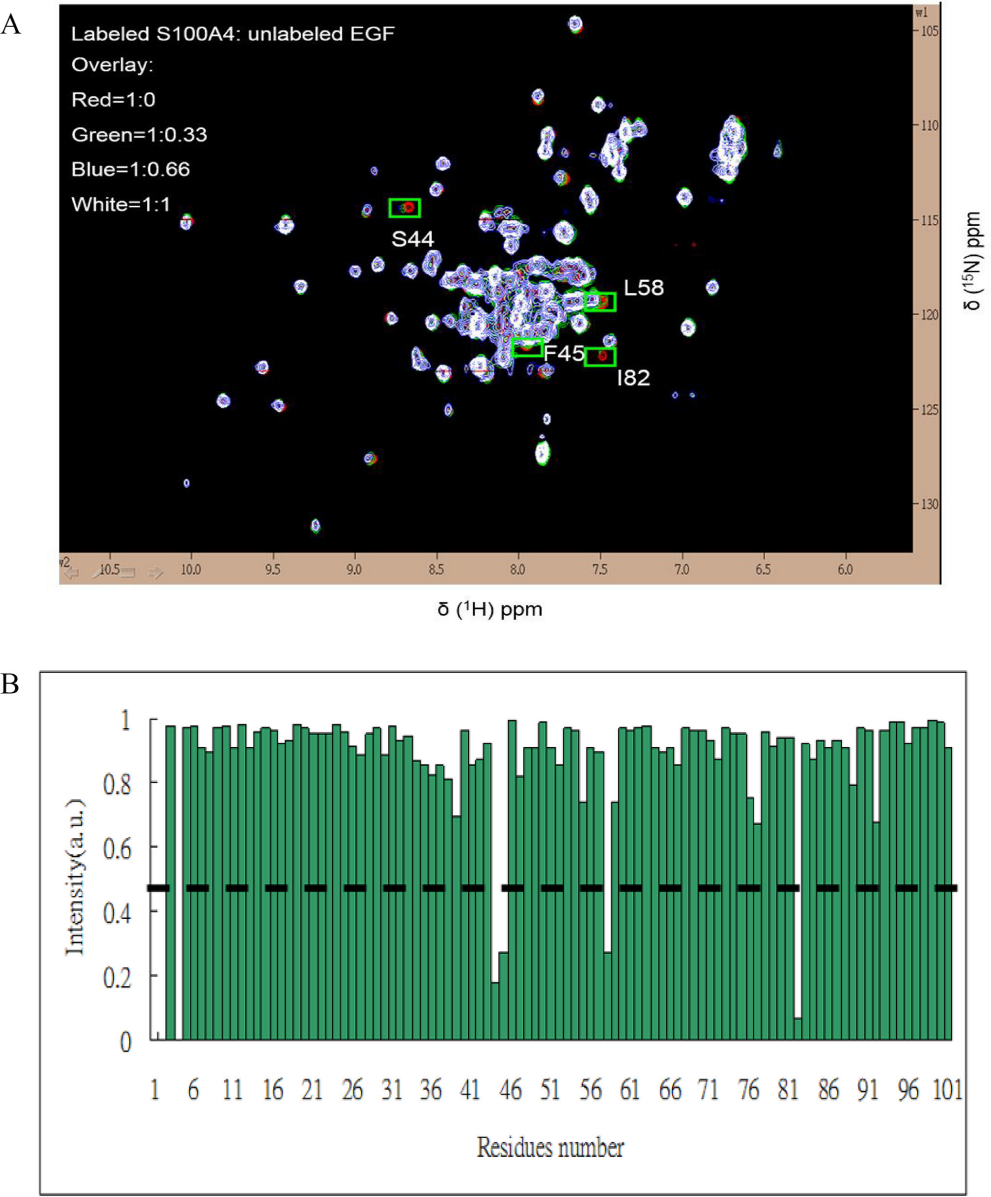
Analysis of the ^1^H-^15^N HSQC Spectra of mS100A4 in Complex with EGF. (A) Overlaid ^1^H-^15^N HSQC spectra of 0.5 mM ^15^N-labeled free mS100A4 (red) and mS100A4: EGF, molar ratio = 1:0.3 (green), 1:0.6 (blue), and 1:1 (white), with the intensity drop indicated by green boxes. (B) Bar graph representing the changes in the cross-peak intensities (I/I_0_) of free mS100A4 and mS100A4 in complex with EGF versus the mS100A4 residue number (1–101). In this plot, (I) represents the cross-peak intensity of mS100A4 in complex with EGF, and (I_0_) represents the initial intensity of free mS100A4. The black dashed line indicates the threshold of selected residues that exhibited a significant decrease in intensity.

The residues of the binding interface between mS100A4 and EGF were recognized by observing the perturbations and intensity changes of chemical shifts in the ^1^H-^15^N HSQC spectrum of free mS100A4 in comparison to that of mS100A4 in complex with EGF. The overlapped HSQC spectra of free mS100A4 and mS100A4 in complex with unlabeled EGF are shown in Fig 2A. We observed a significant decrease in the intensity of the cross peaks in the ^1^H-^15^N HSQC spectrum for mS100A4 with EGF (molar ratio of 1∶1). The residues of the binding interface were identified based on the intensity changes of the HSQC cross peak. A comparison of free mS100A4 (I_0_) with the mS100A4-EGF complex (I) is shown in the bar diagram in Fig 2B.

**Fig 3:**
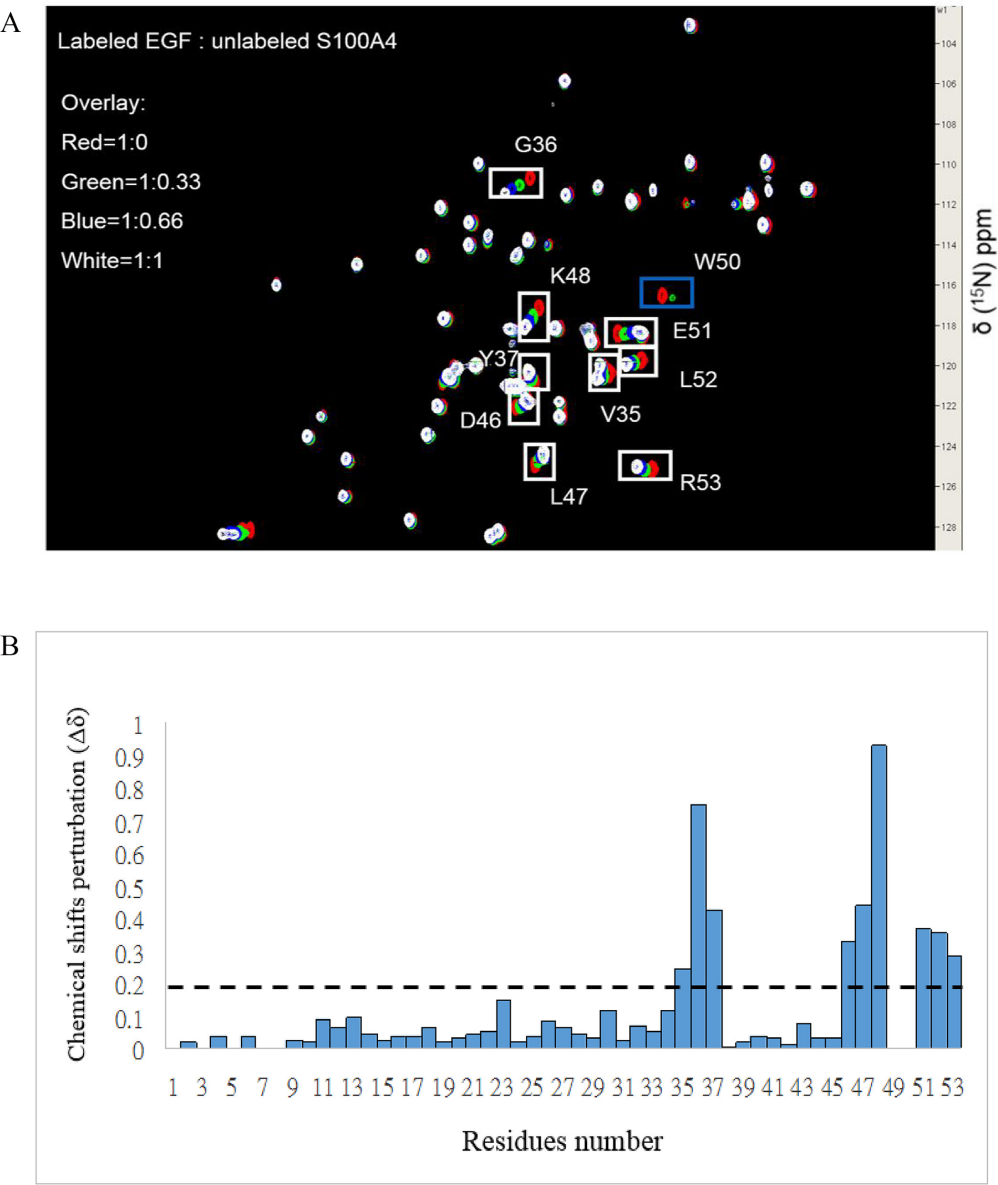
Analysis of the ^1^H-^15^N HSQC Spectra of EGF in Complex with mS100A4. (A) Overlaid ^1^H-^15^N HSQC spectra of 0.5 mM ^15^N-labeled free EGF (red) and EGF: mS100A4, molar ratio = 1:0.3 (green), 1:0.6 (blue), and 1:1 (white), with cross-peak perturbation changes indicated by white boxes and the intensity drop indicated by blue boxes. (B) The weighted average of the chemical shift (^15^N and ^1^H) variations Δδ = [(δ^1^HN)^2^ + 0.2 (δ^15^N)^2^]^1/2^ of residues in EGF on complex formation with mS100A4. The horizontal line is an arbitrary line drawn to demarcate residues that exhibit significant chemical shift perturbations (>0.20 ppm).

These results demonstrated that most of the residues that exhibited a reduction in intensity (S44, F45, L58, and I82) were distributed in loop 2 (residues 42–49), helix 3 (residues 51–63), and helix 4 (residues 72–89). In reversed HSQC titrations, we used ^15^N-labeled EGF with unlabeled mS100A4 at a ratio of 1∶1 and observed the residues of EGF that interacted with mS100A4. Some residues that revealed significant perturbations in the cross peak of EGF during mS100A4 binding, such as V35, G36, Y37, D46, L47, K48, E51, L52, R53, W49, and W50, disappeared (Fig 3A). The bar diagram of the perturbation in the HSQC cross peak of free EGF (I_0_) compared to that of the EGF-mS100A4 complex (I) was used to identify the residues involved in the interface of EGF with mS100A4 (Fig 3B). The observed spectral changes revealed the fast to intermediate exchange of binding between mS100A4 and EGF.

### Structural model of the mS100A4-EGF complex

**Fig 4:**
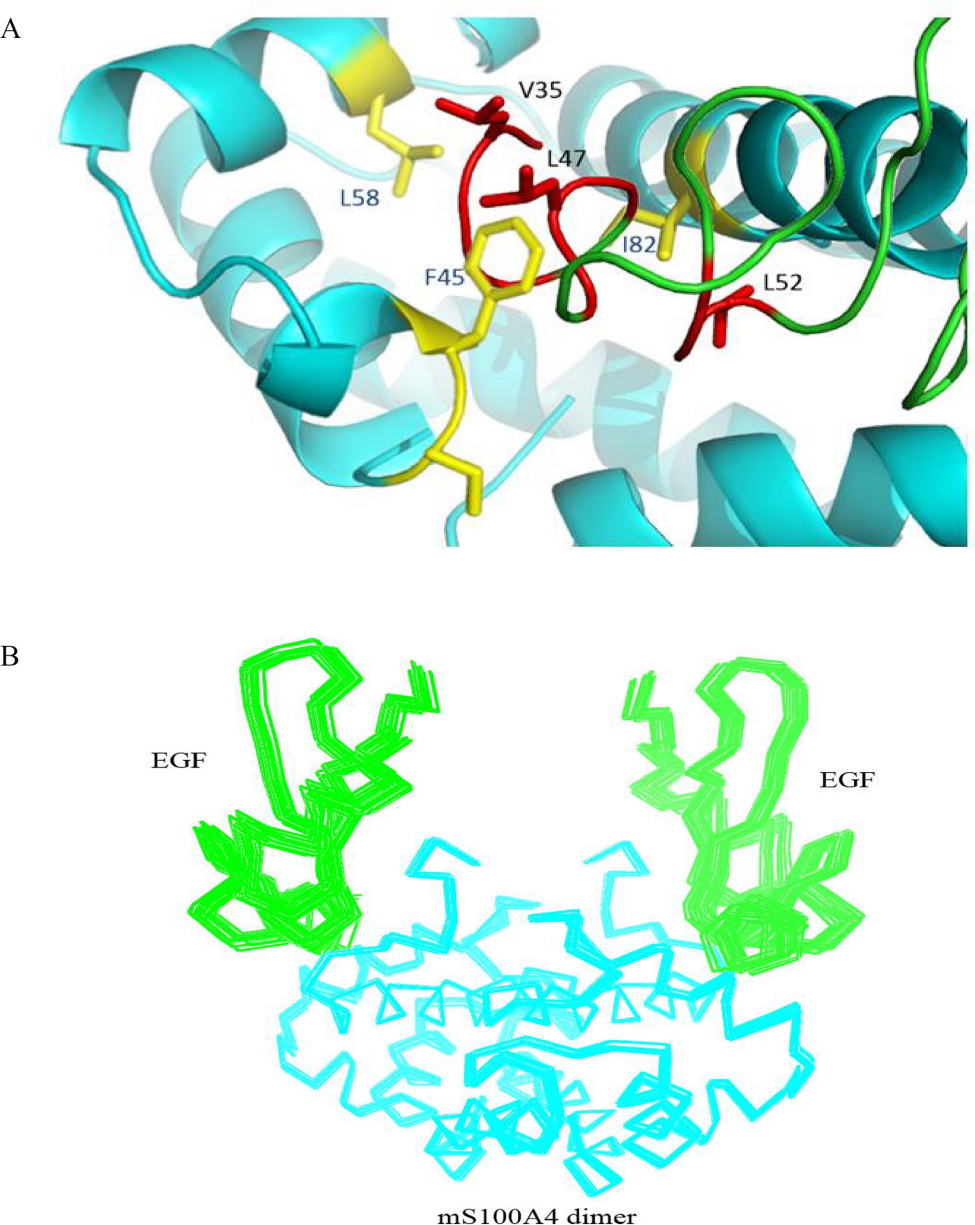
Model of the mS100A4-EGF Complex Determined using HADDOCK. (A) The interface between mS100A4 and EGF. The hydrophobic residues of mS100A4 are yellow, and the hydrophobic residues of EGF are red. (B) Ribbon representation of the 20 best mS100A4-EGF complex overlap, with EGF as green. The mS100A4 homodimer is cyan.

The interface residues were mapped based on the results of the HSQC titrations, and we then generated a model of the mS100A4-EGF complex to describe the interactions between these two proteins. We obtained ambiguous interaction restraints (AIRs) from the cross peak perturbations and decreased intensity of the affected residues in the HSQC titration experiments. Most of these residues were clustered in the same region and formed a single binding interface between mS100A4 and EGF. The NMR restraints allowed HADDOCK to calculate the binary complex model of mS100A4 and EGF. The NMR coordinates of mS100A4 and EGF were taken from the Protein Data Bank. The 2,000 rigid body-docking models were executed using HADDOCK and induced optimized potentials for liquid simulation (OPLSX) parameters. Two hundred lower energy structures were optimized by water refinement and used for analysis. The 200 structures were divided into six clusters, with an RMSD of 0.6 ± 0.3 Å for the overall lowest energy structure. Fig 4A shows the interface of mS100A4-EGF. The hydrophobic residues F45, L58, and I82 of mS100A4 interact with V35, L47, and L52 of EGF. Fig 4B shows the 20 lowest energy structures of the binary mS100A4-EGF model. PROCHECK analysis of the structure indicated good stereochemistry for the structure. In Ramachandran plot statistics, the disallowed region was only 1.1%. The overall average of G-factors was 0.14, which is in the typical region (S1 **Table**).

### Mapping the binding interface between amlexanox and mS100A4

**Fig 5:**
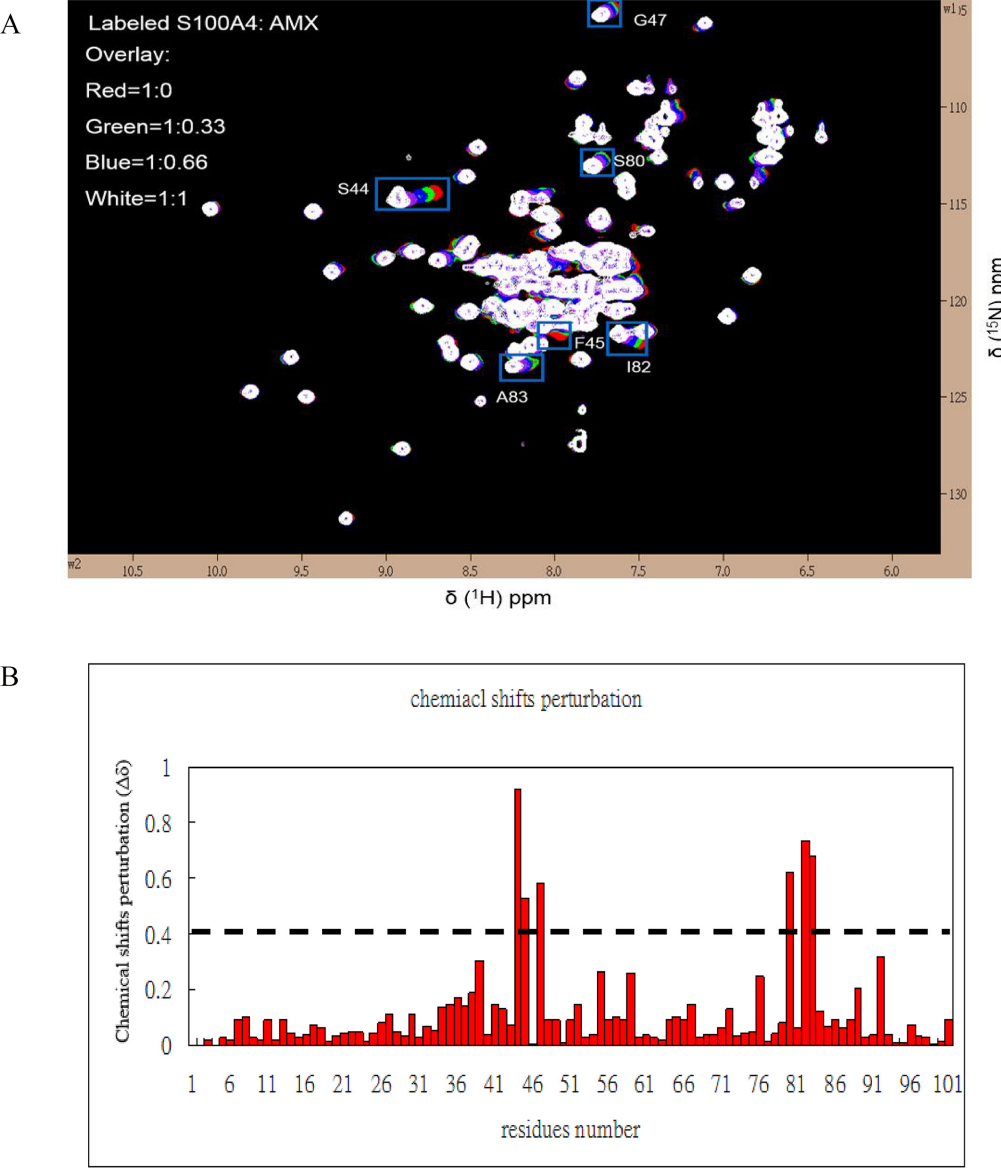
Analysis of the ^1^H-^15^N HSQC Spectra of mS100A4 in Complex with Amlexanox. (A) Overlaid ^1^H-^15^N HSQC spectra of 0.5 mM ^15^N-labeled free mS100A4 (red) and mS100A4: EGF, molar ratio = 1:0.3 (green), 1:0.6 (blue), and 1:1 (white), with the intensity drop indicated by blue boxes. (B) The weighted average of the chemical shift (^15^N and ^1^H) variations Δδ = [(δ^1^HN)^2^ + 0.2 (δ^15^N)^2^]^1/2^ of residues in mS100A4 on complex formation with amlexanox. The horizontal dashed line is an arbitrary line drawn to demarcate residues that exhibit significant chemical shift perturbations. (C) Ribbon representation of mS100A4, with residues that exhibited a decrease in cross-peak intensity mapped (red) on the ribbon diagram.

We used a set of ^1^H-^15^N HSQC titrations to identify the critical residues of the interface between mS100A4 and amlexanox. When comparing the ^1^H-^15^N HSQC spectra of free mS100A4 and mS100A4 in complex with amlexanox, the residues with significant chemical shift perturbations were S44, F45, G47, S80, I82, and A83 (Fig 5A). These residues were distributed in the loop 2 and helix 4 regions, which are the hydrophobic regions of mS100A4. The bar graph of the perturbation in the HSQC cross peak of free mS100A4 (I_0_) compared with that of the mS100A4 complex with amlexanox (I) was used to identify the residues involved in the interface of EGF with mS100A4 (Fig 5B).

### Structural model of the mS100A4–amlexanox complex

**Fig 6:**
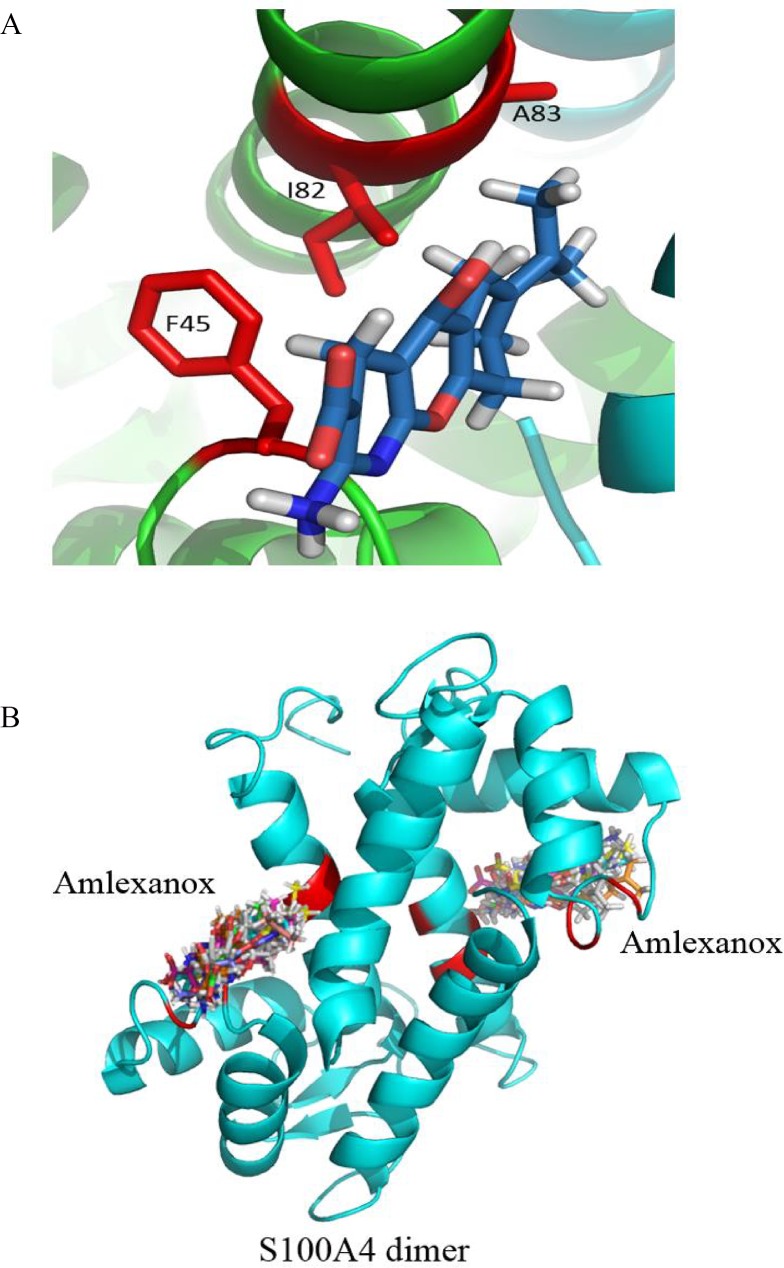
Model of the mS100A4-amlexanox Complex Determined using HADDOCK. (A) The interface between mS100A4 and amlexanox. The hydrophobic residues of mS100A4 are colored red. (B) Ribbon representation of the mS100A4-EGF complex, with 10 overlaid amlexanox structures. The mS100A4 homodimer is blue.

We used the restraints to calculate the mS100A4-amlexanox model using HADDOCK. The obtained AIRs were consistent with the significant chemical shift perturbations of residues in the ^15^N-^1^H HSQC titration experiment. The amlexanox-binding region was situated in the hydrophobic cleft of mS100A4. The hydrophobic interactions of amlexanox interacted with F45, I82, and A83 of mS100A4. The docking results demonstrated that the interaction between amlexanox and mS100A4 was mediated by hydrophobic interactions (Fig 6A). Moreover, we generated the lowest-energy model of the mS100A4-amlexanox complex using HADDOCK. Fig 6B shows the 10 overlapped water refinement structures that were selected from the clusters. After PROCHECK analysis, in Ramachandran plot statistics, the disallowed region was 0.0%. The overall average of G-Factors was -0.27, which is in the typical region (S2 **Table**).

### Functional studies of mS100A4 with EGF and amlexanox

**Fig 7:**
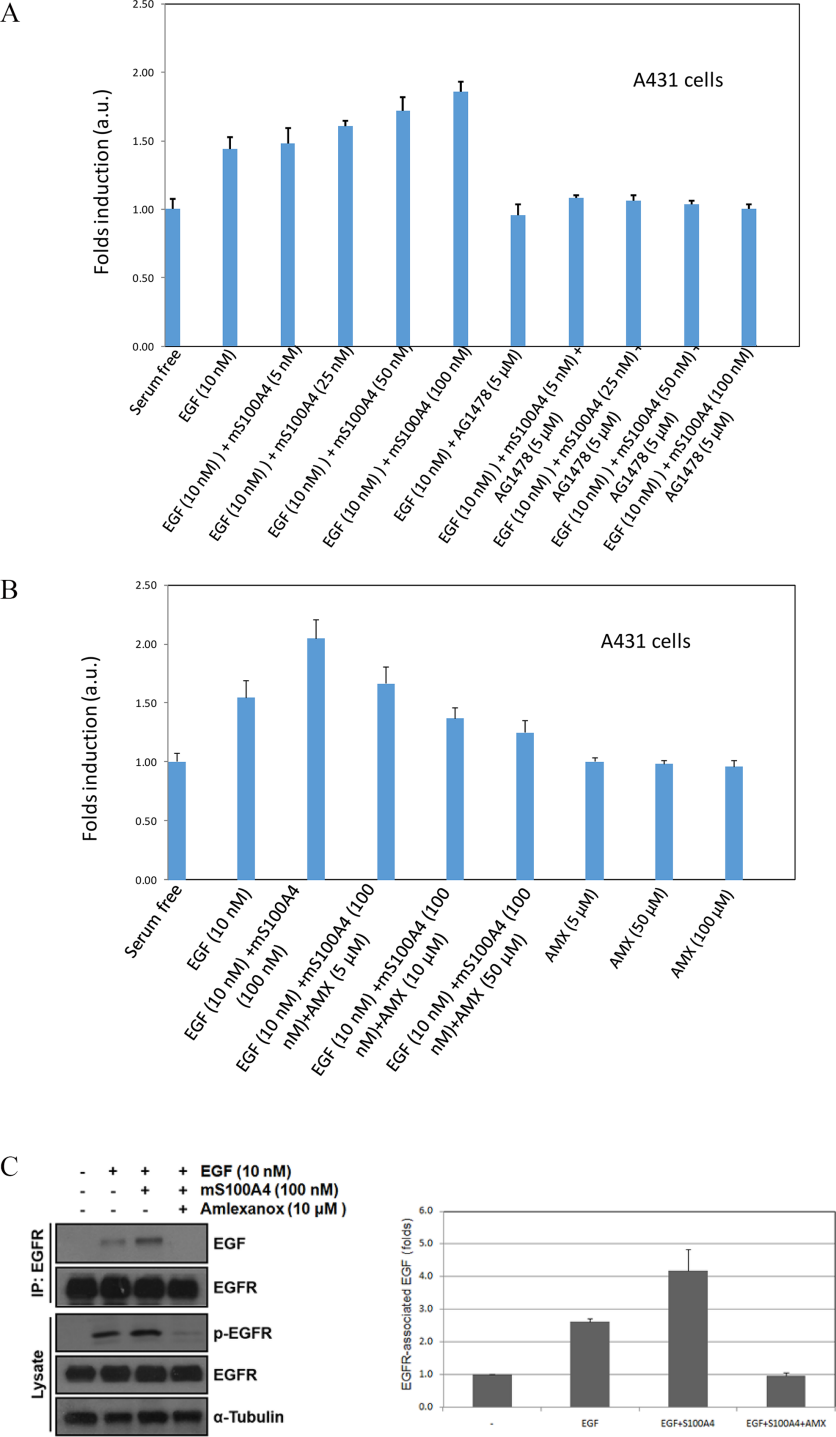
Functional Assay. (A) A431 cells were treated with 10 nM EGF, 10 nM EGF + 5 μM mS100A4, 10 nM EGF + 25 μM mS100A4, 10 nM EGF + 50 μM mS100A4, or 10 nM EGF + 100 μM mS100A4, and cell proliferation was assessed using the WST-1 assay. The relative cell counts after treatment with mS100A4 are plotted as the fold induction, with serum-free medium and AG1478 as the controls (lanes 7–11). The data are expressed as the mean ± SD of 3 independent experiments. (B) Effects of amlexanox on mS100A4-mediated cell proliferation and EGFR signaling. A431 cells were treated with 10 nM EGF, 10 nM EGF + 100 μM mS100A4, 10 nM EGF + 100 μM mS100A4 + 5 μM amlexanox, 10 nM EGF + 100 μM mS100A4 + 10 μM amlexanox, or 10 nM EGF + 100 μM mS100A4 + 50 μM amlexanox. Cell proliferation was analyzed after 48 h. (C) Left panel: A431 cells were serum starved for 24 h and then incubated with or without 10 nM EGF in the presence or absence of 100 nM mS100A4 or 1 μM amlexanox for 1 h. The cell lysate was extracted from each treatment, and 1 mg of cell lysate was immunoprecipitated with an anti-EGFR antibody (Biovision). The amounts of immunoprecipitated EGF and EGFR were examined by Western blotting (upper plot). The amounts of phosphorylated and total EGFR in cell lysate were subsequently detected by Western blotting. Alpha-tubulin was used as an internal control (lower plot). Right panel: The quantitative result of EGFR-associated EGF was shown from two independent experiments.

To explore the downstream effects during exogenous human mS100A4-EGF-mediated bioactivity, we used a WST-1 assay to determine the ability of cell proliferation. Cell proliferation could be affected by the interaction between mS100A4 and EGF. A431 cells are breast carcinoma cells that express endogenous functional EGFR. We observed that low concentrations of EGF (1, 5, and 10 nM) stimulated A431 cell growth in a dose-dependent manner, while high concentrations of S100A4 (50 and 100 nM) slightly promoted cell proliferation (S5 Fig). To determine the effect of S100A4 in EGF-stimulated cell growth, the cells were treated with or without 10 nM EGF in the presence or absence of increased concentrations of mS100A4 (5, 25, 50, and 100 nM). As shown in Fig 7A, we observed an increase in cell proliferation activity after co-treatment with different concentrations of mS100A4. Increasing concentrations of mS100A4 produced a significant induction of EGF-stimulated cell growth (lanes 3–6). AG1478, a kinase inhibitor of EGFR, directly binds to the Tyrosine kinase domain of EGFR, and increases the formation of inactive untethered EGFR dimers [38]. Inhibition of EGFR activity by AG1478 significantly abrogated EGF- and S100A4/EGF-stimulated cell proliferation (lanes 7–11), suggesting that S100A4 enhances EGF-stimulated cell growth might be dominantly through activation of EGF/EGFR pathway. To study the effect of amlexanox on the interaction between mS100A4 and EGF, similar experiments were performed to assess the cell proliferation activity of A431 cell line. When the concentration of amlexanox was increased, the cell proliferation activity decreased, but amlexanox alone had no significant effect on cell proliferation (Fig 7B). Thus, the results suggest that amlexanox inhibited the binding between mS100A4 and EGF and prohibited cell proliferation activity.

To further investigate the effects of the mS100A4-EGF-mediated signaling pathway downstream of the EGFR, we performed an immunoprecipitation assay. Cells treated with EGF alone showed a significant increase in the level of phosphorylated EGFR; nevertheless, after the addition of mS100A4, the EGFR phosphorylation level was higher than that observed in cells treated with EGF alone. Further co-treatment with amlexanox reduced the EGFR phosphorylation level by blocking the interaction of mS100A4 and EGF, reducing EGFR receptor activity (Fig 7C). These results demonstrated that mS100A4 can induce EGFR receptor signaling mediated by the interaction with EGF, and that amlexanox can block the interaction between mS100A4 and EGF in A431 cells.

## Discussion and Conclusions

S100 proteins are associated with a large number of functions, including intra- and extracellular functions, in several cell types. EGFR is one of the primary extracellular receptors that mediate these effects. S100 proteins induced cell growth in EGF-expressing cells. In the current study, based on fluorescence measurements, we defined the dissociation constant (Kd) between human mS100A4 and EGF as 5.2 μM and confirmed the formation and stability of this protein complex. NMR HSQC titrations identified the critical residues in the interface between mS100A4 and EGF. Furthermore, the results were mapped onto the complex and revealed the region comprised by loop-2, helix-3, and helix-4 as the mS100A4-binding interface. In the binding site, the hydrophobic residues F45, L58, and I82 of mS100A4 were associated with the hydrophobic residues V35, L47, and L52 of EGF, respectively, via hydrophobic interactions. According to the results, we elucidated a binary complex model of mS100A4–EGF.

The EGF-binding region in mS100A4 is located in loop-2 and helix-4. We determined that the binding region of amlexanox in mS100A4 is similar to that of EGF. The residues S44, S45, and I82 are the amlexanox-binding sites of mS100A4 involved in the mS100A4−EGF binding interface. In HSQC competition experiments (S4 Fig), EGF and amlexanox competed for binding to S100A4. We observed that when amlexanox was added to the S100A4 protein (molar ratio of 1:1), the addition of EGF did not cause significantly chemical shift perturbations in S100A4. This finding suggested that amlexanox could block EGF-mutant S100A4 binding.

Previous study has shown that low concentration (< 3 nM) of EGF stimulates cell growth, but high concentration (>3 nM) of EGF inhibits cell growth of A431 cells [39]. However, it has also been demonstrated that EGF stimulates cell proliferation in a dose-dependent manner (1, 3, and 30 ng/ml; i.e. approximately 0.16, 0.5, and 5 nM) in accordance with EGF-stimulated phosphorylation amounts of EGFR in A431 cells, which are more sensitive to a EGFR tyrosine kinase inhibitor, Gefitinib (Iressa) [40]. Although the Kd for the EGF-S100A4 interaction *in vitro* was determined to be ~5 μM in our current study, the buffer condition of the *in vitro* Kd measurement was quite different in that of cell culture medium. In addition, the physiological concentration of S100A4 is in the nanomolar range [41]. Therefore, we used the concentrations of S100A4 with nanomolar range in the cell proliferation assay. Our results demonstrated that EGF stimulates cell proliferation of A431 cells depending on the concentrations (1, 5, and 10 nM) of EGF in serum-free medium containing 0.1% BSA, while high concentrations of S100A4 (50 and 100 nM) also slightly promotes cell proliferation (S5 Fig). Therefore, we can’t exclude that S100A4 might stimulate cell proliferation in EGF-independent pathway. It has also been known that S100A4 interacts with Receptor for Advanced Glycation Endproducts (RAGE), which contributes to metastatic property [42]. In Fig 7A, addition of AG1478, an EGFR kinase inhibitor, significantly reduced EGF or EGF/S100A4-induced cell proliferation, suggesting that S100A4 stimulates cancer cell proliferation, at least in part, through enhancing EGF/EGFR pathway. According to the WST-1 assay results (Fig 7B), cells treated with EGF alone exhibited a significant increase in cell proliferation. However, when mS100A4 was added, the cell proliferation increased one-fold. Further co-treatment with amlexanox reduced cell proliferation because amlexanox blocks the interaction of mS100A4 and EGF. In the co-immunoprecipitation assay, we used an EGF antibody to detect EGF bound to EGFR. The results indicated that when the cells are treated with EGF alone, the detectable EGF interacting with EGFR increases (Fig 7C, second lane). The third lane in Fig 7C showed that S100A4 induces the binding of EGF to EGFR. With conducted further co-treatment with amlexanox, and found that the detectable level of EGF binding to EGFR decreased (Fig 7C, fourth lane). These results indicate that S100A4 can help EGF associate with the EGFR, and that amlexanox can block the interaction of mS100A4 with EGF, which is crucial for reducing EGFR pathway activity. This effect indicated that amlexanox may be a potential antagonist of the EGFR and may be useful for the development of new antagonists for mS100A4 and EGF.

## Supporting Information

S1 Fig Properties of the EGF Protein. (A) SDS PAGE of EGF. (B) ESI-MASS of EGF. (C) Protein sequence of EGF.
(TIF)

S2 Fig Properties of the Mutant S100A4 Protein. (A) SDS PAGE of mutant S100A4. (B) ESI-MASS of mutant S100A4. (C) Protein sequence of mutant S100A4.
(TIF)

S3 Fig Structure Formula of Amlexanox (TIF)

S4 Fig HSQC Competition of EGF and Amlexanox Competing for Binding to Mutant S100A4; the Interface Residues between Mutant S100A4 with Amlexanox are Boxed in White. According to this result, when amlexanox was added to S100A4 protein (a ratio of 1:1), EGF did not cause significant chemical shifts for the perturbation of S100A4.
(TIF)

S5 Fig A431 cells were treated with 1 nM EGF, 5 nM EGF, 10 nM EGF, 25 nM mS100A4, 50 nM mS100A4, 100 nM mS100A4, and cell proliferation was assessed using the WST-1 assay. The relative cell counts after treatment with mS100A4 are plotted as the fold induction, with serum-free medium and AG1478 as the controls (lanes 7–11). The data are expressed as the mean ± SD of 3 independent experiments. Cell proliferation was analyzed after 48 h.
(TIF)

S1 Table PROCHECK Results of mS100A4- EGF Complex. (TIF)

S2 Table PROCHECK Results of mS100A4- AMX Complex. (TIF)
