# Supplementary figures and images for: Amlexanox Blocks the Interaction between S100A4 and Epidermal Growth Factor and Inhibits Cell Proliferation
Source: PLoS One. 2016 Aug 25;11(8):e0161663. doi: 10.1371/journal.pone.0161663 (PMC4999211; doi:10.1371/journal.pone.0161663)

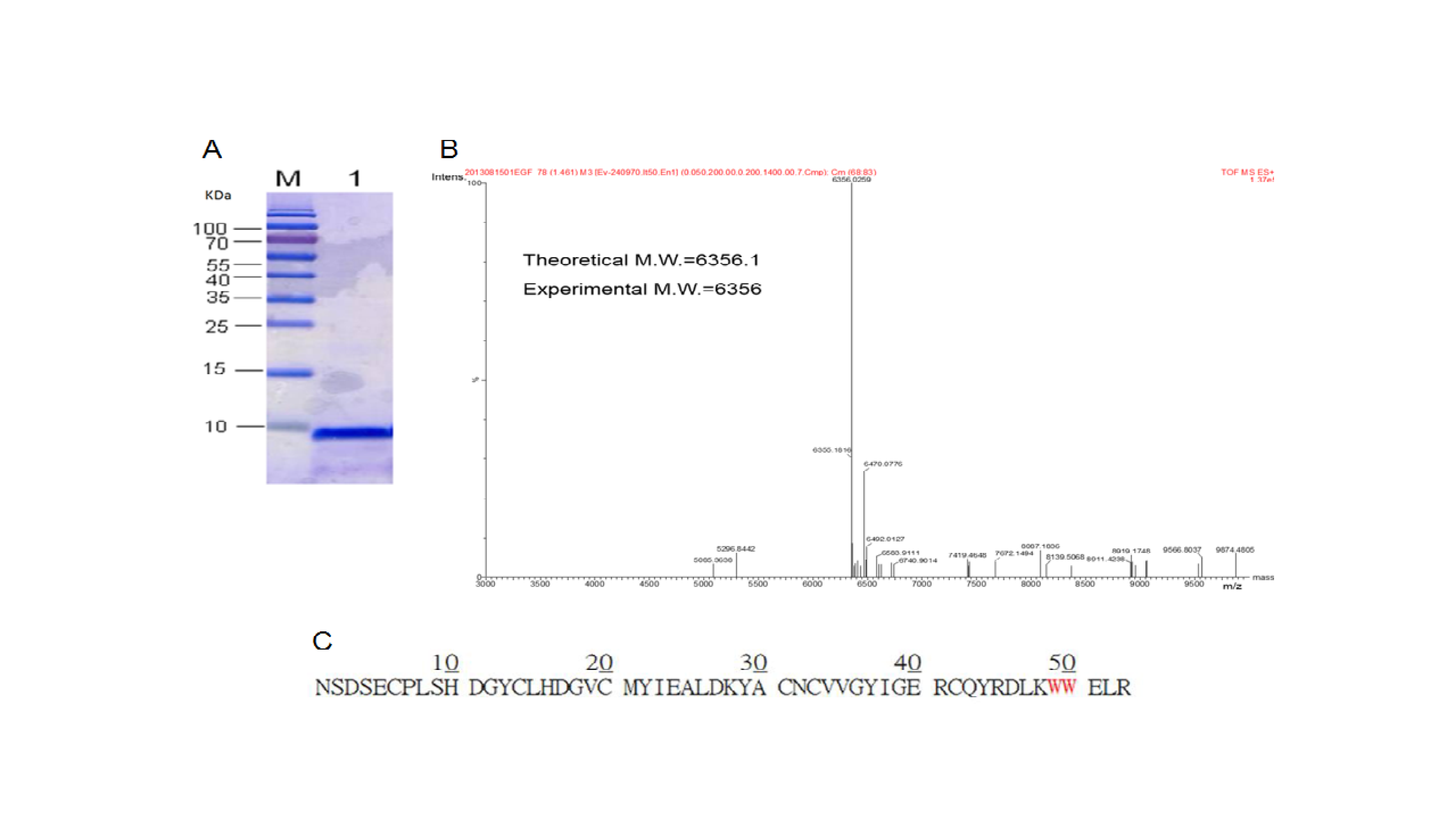

Supplement: S1 Fig — (A) SDS PAGE of EGF. (B) ESI-MASS of EGF. (C) Protein sequence of EGF. (TIF) [file pone.0161663.s001.tif]

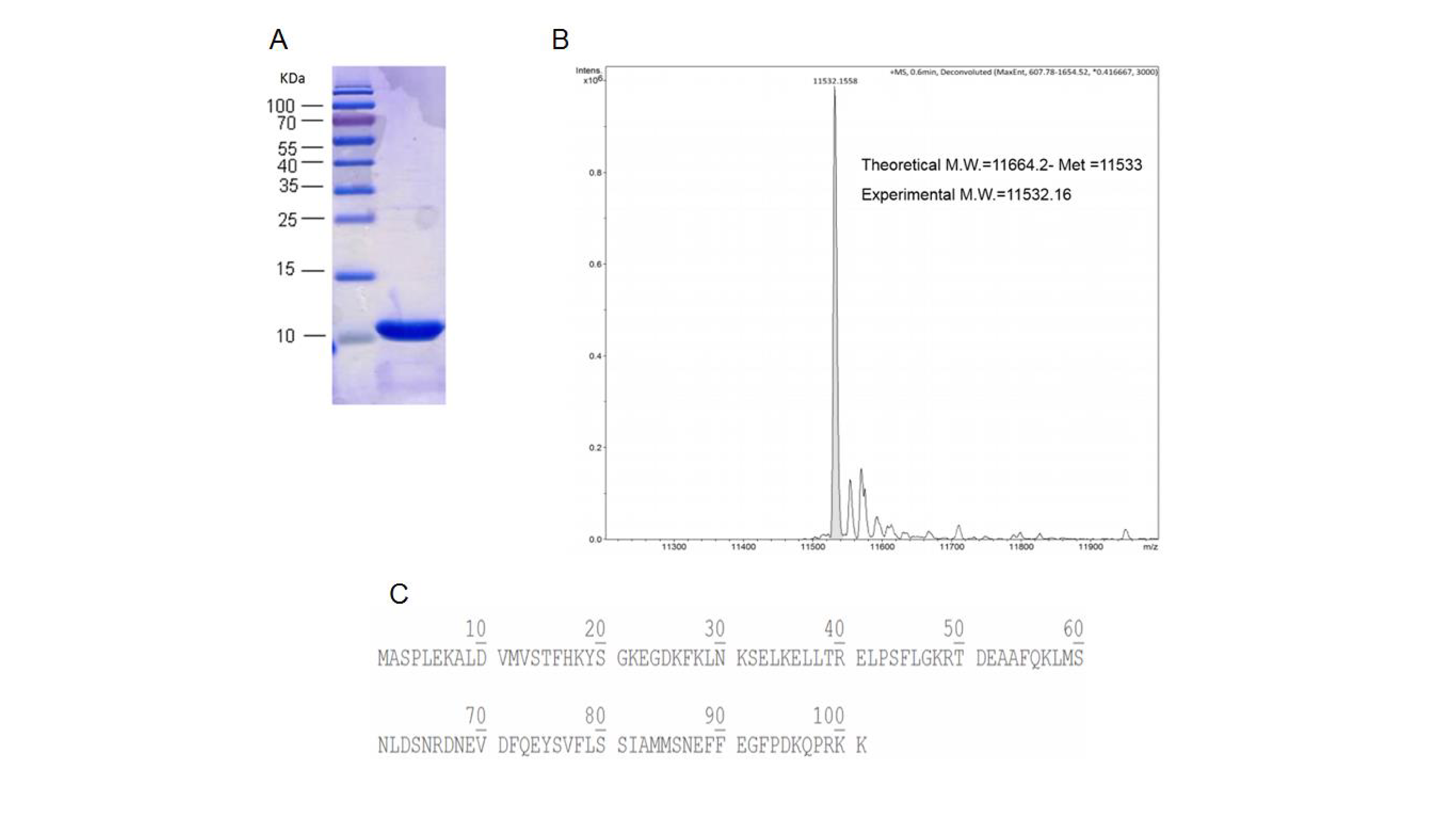

Supplement: S2 Fig — (A) SDS PAGE of mutant S100A4. (B) ESI-MASS of mutant S100A4. (C) Protein sequence of mutant S100A4. (TIF) [file pone.0161663.s002.tif]

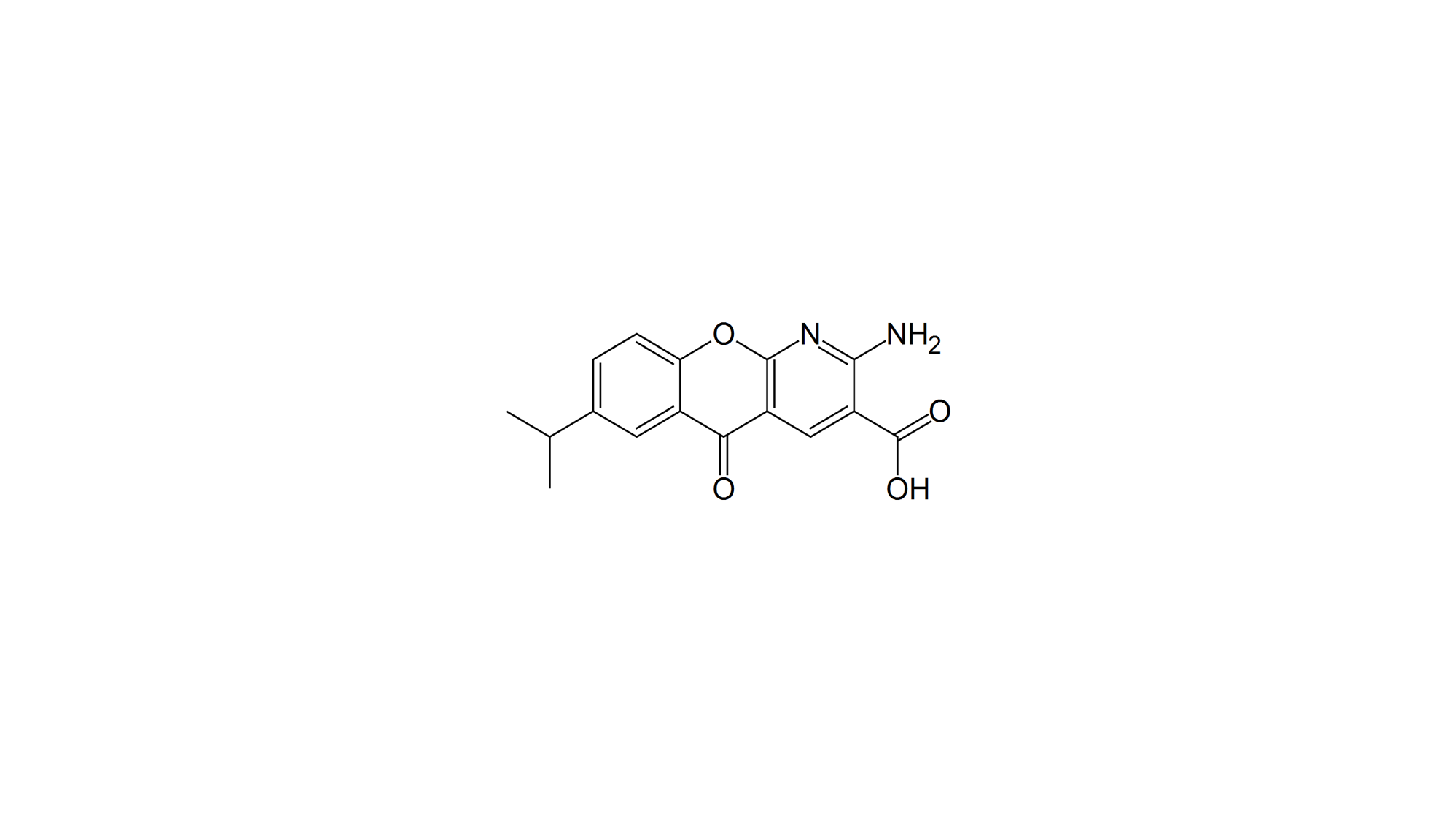

Supplement: S3 Fig — (TIF) [file pone.0161663.s003.tif]

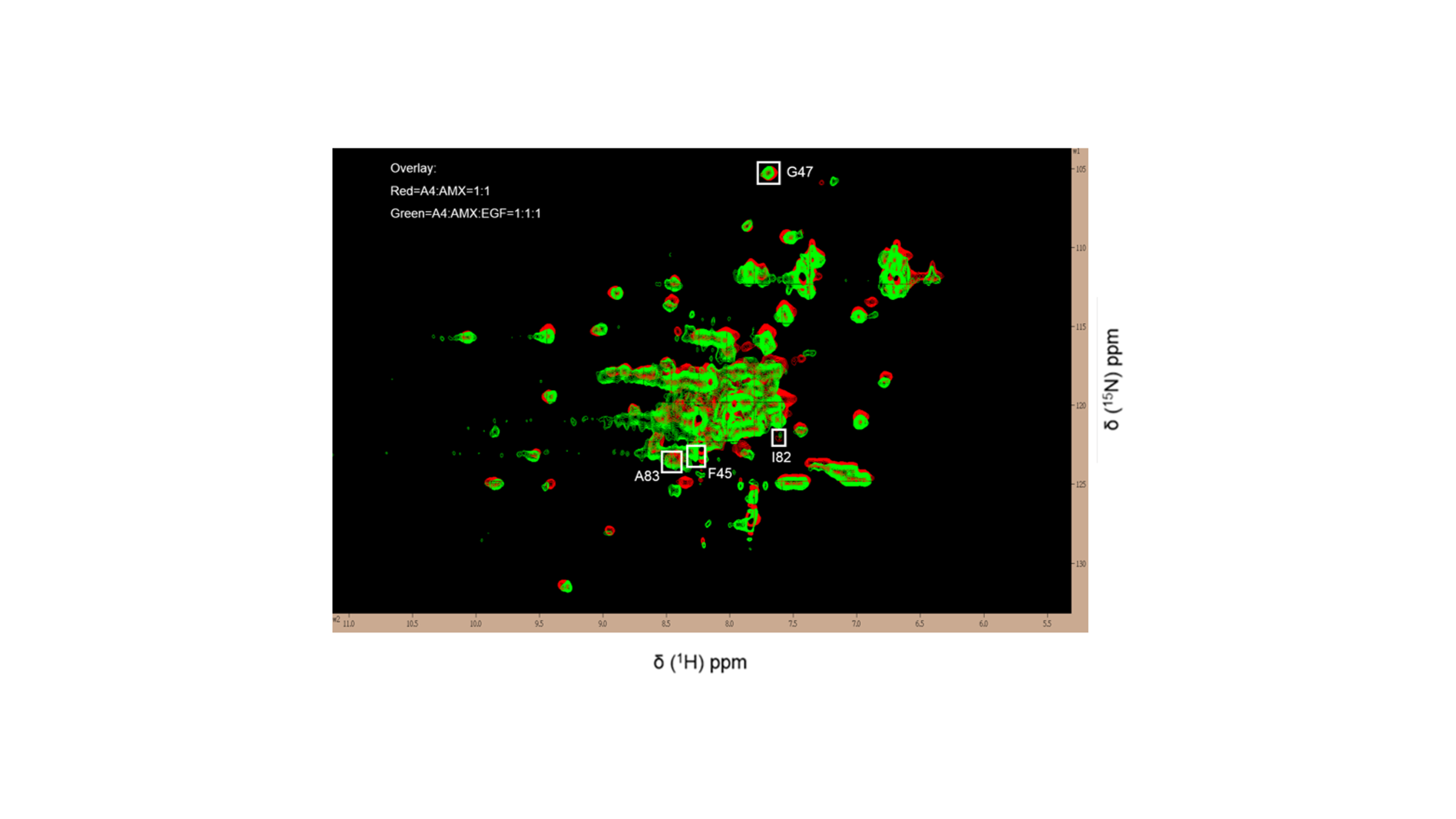

Supplement: S4 Fig — According to this result, when amlexanox was added to S100A4 protein (a ratio of 1:1), EGF did not cause significant chemical shifts for the perturbation of S100A4. (TIF) [file pone.0161663.s004.tif]

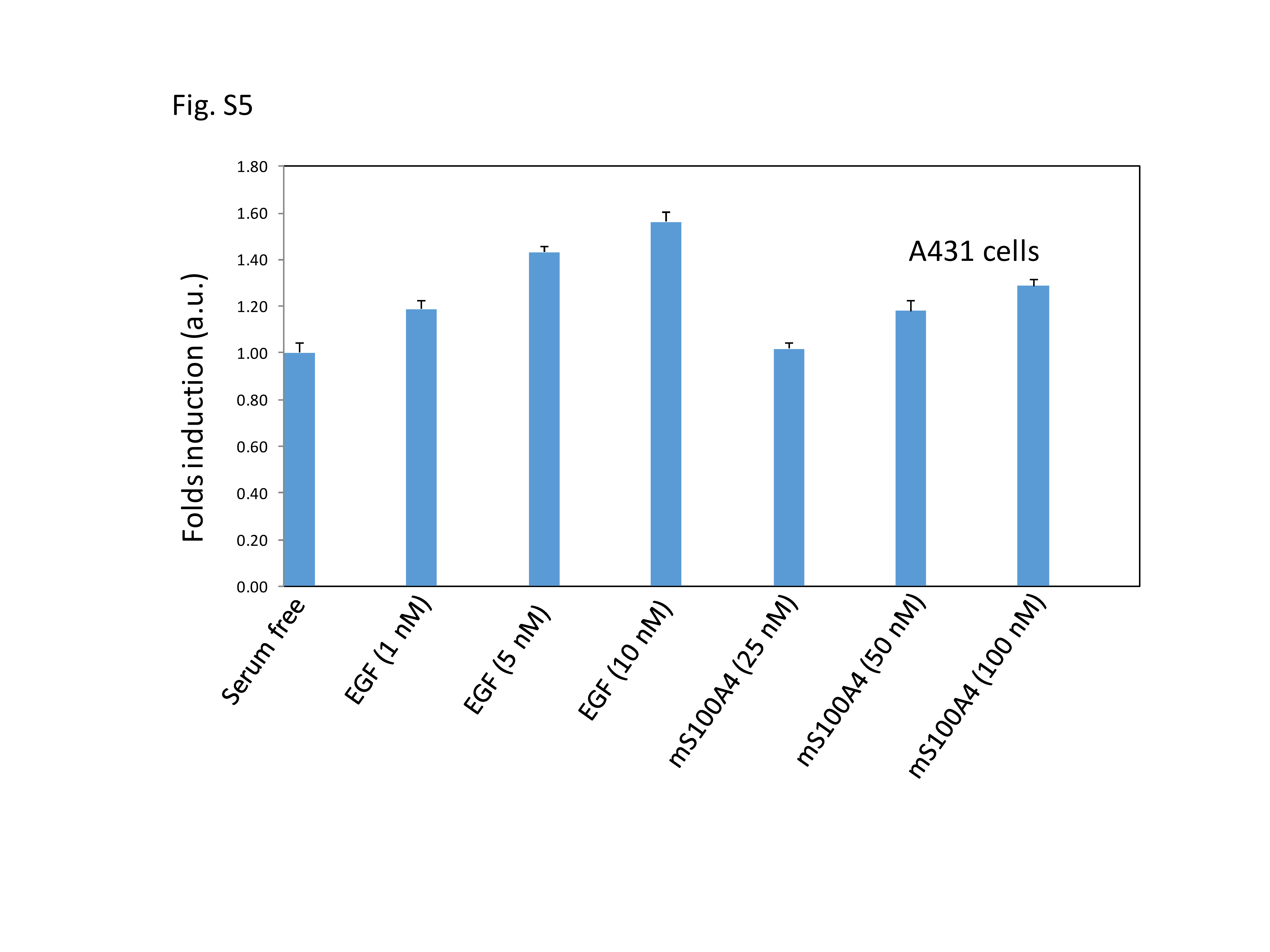

Supplement: S5 Fig — The relative cell counts after treatment with mS100A4 are plotted as the fold induction, with serum-free medium and AG1478 as the controls (lanes 7–11). The data are expressed as the mean ± SD of 3 independent experiments. Cell proliferation was analyzed after 48 h. (TIF) [file pone.0161663.s005.tif]

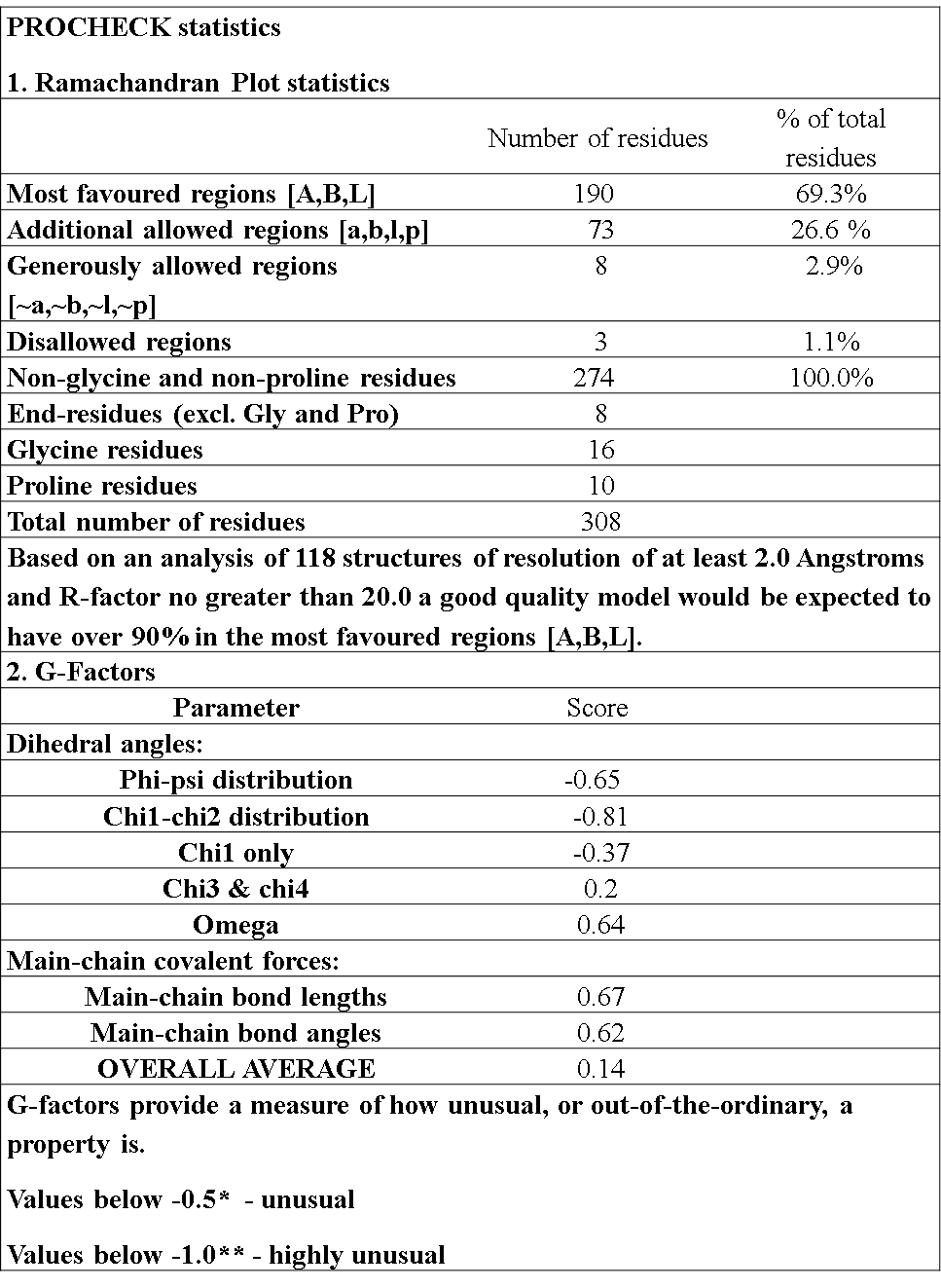

Supplement: S1 Table — (TIF) [file pone.0161663.s006.tif]

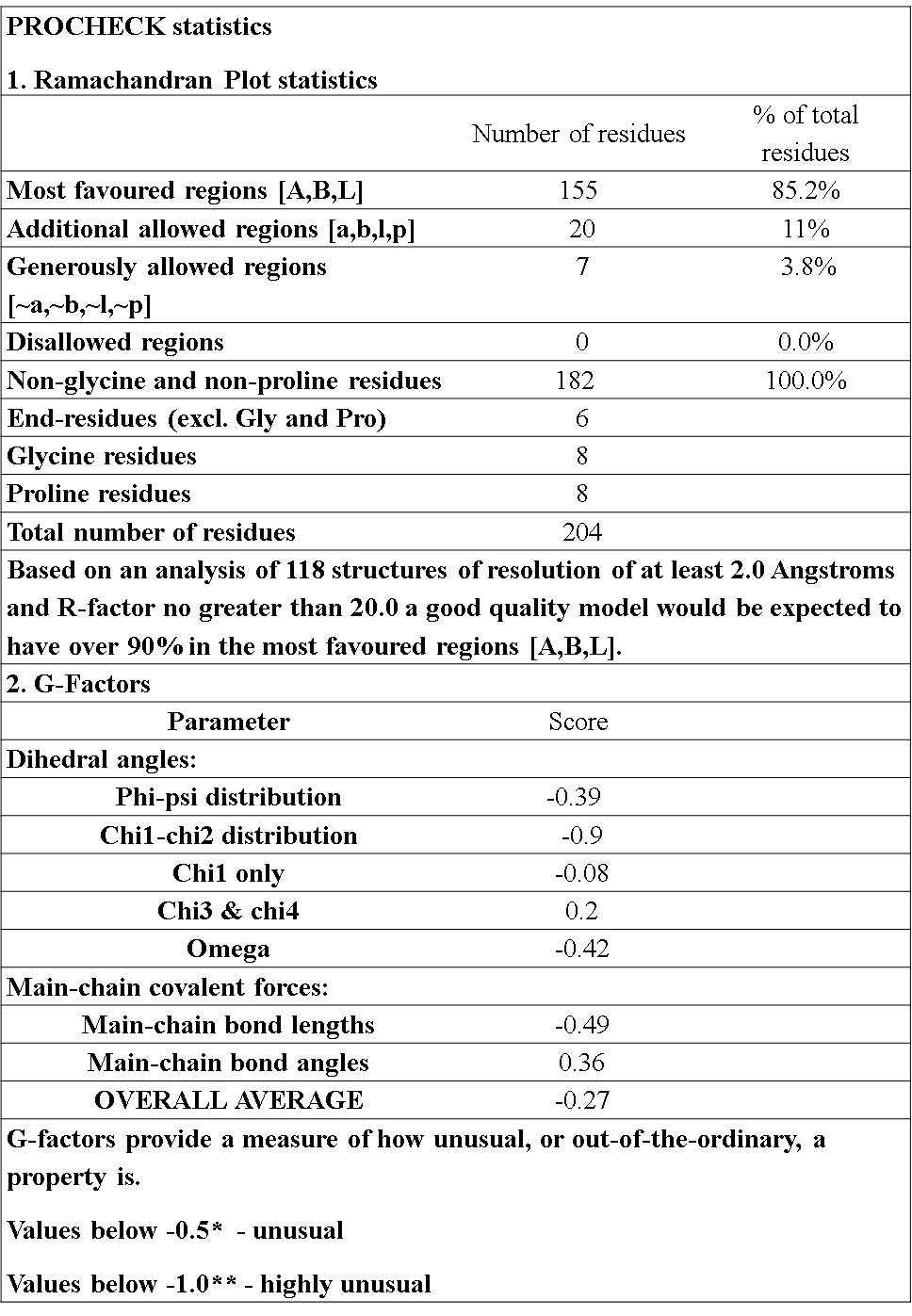

Supplement: S2 Table — (TIF) [file pone.0161663.s007.tif]
